# Oxytocin Impairs the Recognition of Micro-Expressions of Surprise and Disgust

**DOI:** 10.3389/fpsyg.2022.947418

**Published:** 2022-06-29

**Authors:** Qi Wu, Yanni Xie, Xuanchen Liu, Yulong Liu

**Affiliations:** ^1^Department of Psychology, School of Educational Science, Hunan Normal University, Changsha, China; ^2^Cognition and Human Behavior Key Laboratory of Hunan Province, Hunan Normal University, Changsha, China; ^3^School of Finance and Management, Changsha Social Work College, Changsha, China

**Keywords:** oxytocin, micro-expression, micro-expression recognition, macro-expression, disgust, surprise

## Abstract

As fleeting facial expressions which reveal the emotion that a person tries to conceal, micro-expressions have great application potentials for fields like security, national defense and medical treatment. However, the physiological basis for the recognition of these facial expressions is poorly understood. In the present research, we utilized a double-blind, placebo-controlled, mixed-model experimental design to investigate the effects of oxytocin on the recognition of micro-expressions in three behavioral studies. Specifically, in Studies 1 and 2, participants were asked to perform a laboratory-based standardized micro-expression recognition task after self-administration of a single dose of intranasal oxytocin (40 IU) or placebo (containing all ingredients except for the neuropeptide). In Study 3, we further examined the effects of oxytocin on the recognition of natural micro-expressions. The results showed that intranasal oxytocin decreased the recognition speed for standardized intense micro-expressions of surprise (Study 1) and decreased the recognition accuracy for standardized subtle micro-expressions of disgust (Study 2). The results of Study 3 further revealed that intranasal oxytocin administration significantly reduced the recognition accuracy for natural micro-expressions of surprise and disgust. The present research is the first to investigate the effects of oxytocin on micro-expression recognition. It suggests that the oxytocin mainly plays an inhibiting role in the recognition of micro-expressions and there are fundamental differences in the neurophysiological basis for the recognition of micro-expressions and macro-expressions.

## Introduction

Efficiently and accurately recognizing the facial expressions of others is probably one of the most important perceptual tasks we encounter as social creatures. Without being able to read the face, we are very unlikely to understand the true feelings and intentions of others and thereby coordinate our actions. However, not all our emotions are necessarily revealed by the face. As social creatures, in a number of situations, we all need to conceal or disguise our true emotions to achieve our personal goals (Ekman, 1971). Fortunately, despite the efforts to hide, the concealed emotions may still leak, and those leaked emotions are usually displayed in the form of micro-expressions (e.g., Yan et al., 2013; Matsumoto and Hwang, 2018). Micro-expressions are fleeting facial expressions which reveal the emotion that a person tries to conceal (Ekman and Friesen, 1969; Ekman, 2009; Yan et al., 2013; Matsumoto and Hwang, 2018). They usually occur in high-stake situations, especially for the ones who have something important to gain or lose (Ekman, 2009). The micro-expressions usually resemble the appearances of macro-expressions (i.e., the typical or the “normal” facial expressions, which are produced when emotions occur; Ekman, 2003; Matsumoto and Hwang, 2011, 2018; Yan et al., 2013), but their durations are significantly shorter (a typical micro-expression lasts no longer than 0.5 s, while the macro-expression typically lasts between 0.5 and 4 s; Matsumoto and Hwang, 2011, 2018; Yan et al., 2013). Due to their involuntary nature and their close association with emotion hiding and deception (Ekman and Friesen, 1969; Ekman, 2003, 2009; Matsumoto and Hwang, 2011, 2018; Yan et al., 2013), micro-expressions have great application potentials in fields that require face-to-face interpersonal skills, such as national security, law enforcement, medical treatment, education, and politics (e.g., Russell et al., 2008; Endres and Laidlaw, 2009; Frank et al., 2009; Stewart et al., 2009; Weinberger, 2010; Matsumoto and Hwang, 2011, 2018; Frank and Svetieva, 2015; Zhu et al., 2017, 2019; Stewart and Svetieva, 2021). However, because the durations of micro-expressions are so short, it is very difficult for observers to accurately detect and recognize these fleeting involuntary facial expressions (e.g., Ekman and Friesen, 1969; Ekman, 2009; Matsumoto and Hwang, 2011; Shen et al., 2012, 2016; Zeng et al., 2018).

The short duration of micro-expression is not the only obstacle we encountered during micro-expression recognition. To the best of our knowledge, only a few studies have tried to investigate the psychological or brain mechanisms of micro-expression recognition, which makes the scientists difficult to design efficient micro-expression recognition training programs (e.g., Ekman, 2002; Frank et al., 2009; Matsumoto and Hwang, 2011; Döllinger et al., 2021). For example, researchers have found that the factors like emotional context, age, childhood family environment, personality, and profession (e.g., Frank et al., 2009; Hurley et al., 2014; Zhang et al., 2014, 2020b; Svetieva and Frank, 2016; Demetrioff et al., 2017; Felisberti, 2018) may affect the recognition accuracy of micro-expressions. Diverse brain areas (e.g., frontal lobes, insula, precuneus, amygdala, cingulate cortex, etc.) are also found to be involved in the recognition of micro-expressions (Zhang et al., 2020a,b). Most importantly, some recent evidence suggests that there are fundamental differences in the neuropsychological basis for the recognition of micro-expressions and macro-expressions. For example, researchers found that the inferior temporal gyrus and widespread regions of frontal lobe are differently involved in the processing of micro-expressions and macro-expressions, and these differences are mainly located in the left hemisphere (Shen et al., 2016). Researchers also found that while the sensorimotor simulation mainly facilitates the recognition of macro-expressions (e.g., Wood et al., 2016), facial mimicry from one’s face was found to disrupt the recognition of micro-expressions, and this detrimental effect was mainly driven by the facial mimicry of the lower face (Wu et al., 2016; Zeng et al., 2018). Recent evidence also showed that contrary to the ubiquitous ingroup advantage in macro-expression recognition (i.e., much easier to recognize the macro-expressions of ingroup members than the macro-expressions of outgroup members), participants actually were easier to recognize the micro-expressions of outgroup members than the micro-expressions of ingroup members (Xie et al., 2019). Such an ingroup disadvantage may even persist after receiving professional trainings in micro-expression recognitions (Xie et al., 2019).

Although the neurophysiological basis of the recognition of micro-expressions seems to be poorly understood, mainstream psychology has long documented the fundamental role of the oxytocin in the perception of macro-expressions (for review, see Leppanen et al., 2017; Romero-Martínez et al., 2021). Oxytocin is a complex, multifunctional neuropeptide that is primarily produced in magnocellular neurons in the paraventricular and supraoptic nuclei of the hypothalamus and released by the posterior pituitary. This evolutionary ancient and conserved neuropeptide affects the central and peripheral nervous systems in both sexes (Carter, 2014; Ma et al., 2016). In the peripheral nervous system, it mainly functions as a hormone which facilitates the process of birth and lactation (Carter, 2014). In the central nervous system, it functions as a neuromodulator which broadly affects the brain regions (e.g., hypothalamus, thalamus, globus pallidus, substantia nigra, caudate, amygdala, and insula) that are implicated in social and affective processes (Ma et al., 2016; Quintana et al., 2021). As for the macro-expression recognition, many studies found that participants displayed higher recognition accuracy after receiving intranasal administration of oxytocin, regardless of the emotional valence of target facial expressions (e.g., Domes et al., 2007; Prehn et al., 2013; Bate et al., 2014; Feeser et al., 2014; Timmermann et al., 2017; Wang et al., 2020). Some studies also suggest that intranasal oxytocin may only specifically enhance the recognition for some of the macro-expressions (e.g., specifically enhance the recognition for macro-expressions like surprise, happiness, anger, disgust, or fear; e.g., Di Simplicio et al., 2009; Marsh et al., 2010; Fang et al., 2014; Shin et al., 2018; Schwaiger et al., 2019), whereas a few studies seem to indicate that oxytocin may inhibits the processing of all negative macro-expressions (e.g., Ellenbogen et al., 2012; Domes et al., 2013; Ma et al., 2020; Bach et al., 2021). The results of meta-analysis provide important support for the emotion-specific account of the effects of oxytocin. They suggest that intranasal oxytocin administration mainly have an emotion-specific enhancement effect on the recognition of happy and fearful macro-expressions (Shahrestani et al., 2013; Leppanen et al., 2017). In sum, although the conclusions are not completely homogeneous, the currently available evidence suggests that the oxytocin system plays an important role in facilitating the recognition of typical facial expressions.

Can oxytocin also affect the recognition of micro-expressions? Answers to this question will allow us to have a better understanding of the mechanisms underlying the micro-expression recognition, and help the researchers to develop better micro-expression recognition tools or training programs. However, no previous study has ever directly investigated this issue. Only some indirect evidence has shed some light on this question. For example, while the main function of oxytocin is to facilitate pair bonding, social adaptation, and trust (e.g., Carter, 2014; Ma et al., 2016; Xu et al., 2019; Kurokawa et al., 2021), intranasal oxytocin administration was found to decrease the lie detection ability for both males and females (Pfundmair et al., 2017). Given the association between micro-expression and deception (Ekman and Friesen, 1969; Ekman, 1971, 2003, 2009; Matsumoto and Hwang, 2011, 2018; Yan et al., 2013), this study further suggests that oxytocin may have a detrimental effect on the recognition of micro-expressions. In addition, previous studies also found that intranasal oxytocin can increase the facial mimicry during the macro-expression perception process (Korb et al., 2016; Pavarini et al., 2019), which also suggest that oxytocin may decrease the recognition accuracy for micro-expressions through the enhancement of disruptive facial mimicry (Wu et al., 2016; Zeng et al., 2018). Therefore, in the present research, we hypothesized that oxytocin may inhibits the recognition of micro-expressions.

As an efficient and noninvasive method to manipulate oxytocin level in the brain, intranasal administration of oxytocin has widely been accepted in the studies of the effects of oxytocin on human behaviors^1^ (e.g., Domes et al., 2007; Prehn et al., 2013; Bate et al., 2014; Ma et al., 2016; Walum et al., 2016; Leppanen et al., 2017; Timmermann et al., 2017; Schwaiger et al., 2019; Wang et al., 2020; Quintana et al., 2021; Romero-Martínez et al., 2021). Therefore, in the present research, we utilized a double-blind, placebo-controlled, mixed-model experimental design to investigate the effects of intranasal oxytocin administration on the recognition of micro-expressions. More specifically, considering previous studies have suggested that oxytocin may have an emotion-specific enhancement effect for the recognition of macro-expressions (e.g., Di Simplicio et al., 2009; Marsh et al., 2010; Leknes et al., 2012; Shahrestani et al., 2013; Fang et al., 2014; Leppanen et al., 2017; Shin et al., 2018; Schwaiger et al., 2019), in the present research, in three behavioral studies we investigated whether intranasal oxytocin has differential effects on the recognition of different categories of micro-expressions (i.e., micro-expressions of the six basic emotions, including sadness, surprise, anger, disgust, fear, and happiness; e.g., Matsumoto and Hwang, 2011; Shen et al., 2012; Hurley et al., 2014; Svetieva and Frank, 2016; Wu et al., 2016; Demetrioff et al., 2017; Zeng et al., 2018; Zhang et al., 2020a). Specifically, in Study 1 and 2, we tested the effects of oxytocin on the recognition of standardized intense (Study 1) and subtle (Study 2) micro-expressions.^2^ In Study 3, we further examined the effects of oxytocin on the recognition of natural micro-expressions. Following previous studies (e.g., Di Simplicio et al., 2009; Prehn et al., 2013; Fang et al., 2014; Feeser et al., 2014; Korb et al., 2016; Shin et al., 2018), we exclusively focused on male samples to reduce complications linked to female menstrual cycle. The computer codes and other materials that were employed for performing these three studies are available at https://osf.io/xebvd/.^3^

## Study 1

Previous researches have suggested that the amplitude of micro-expressions may modulate the effects of facial mimicry on micro-expression recognition (Wu et al., 2016; Zeng et al., 2018). Considering previous researches also suggest that the intranasal oxytocin administration may increase the facial mimicry during the process of facial expression recognition (Korb et al., 2016; Pavarini et al., 2019), these studies further suggest that the intensity of micro-expressions may also moderate the effects of oxytocin during micro-expression recognition (Wu et al., 2016; Zeng et al., 2018). Therefore, in Study 1, we investigated the effects of oxytocin on the recognition of intense micro-expressions at first. Specifically, we employed a laboratory-based paradigm of Japanese and Caucasian Brief Affect Recognition Test (JACBART) to present these micro-expressions (Matsumoto et al., 2000). This well-accepted paradigm presents the micro-expressions in a standardized way which utilizes apparent movement to present the facial dynamics and it can efficiently control the key factors that may moderate the recognition of micro-expressions (e.g., the head movement of the target, the emotion context, the duration and the intensity of micro-expressions; e.g., Matsumoto et al., 2000; Ekman, 2009; Endres and Laidlaw, 2009; Matsumoto and Hwang, 2011; Hurley et al., 2014; Svetieva and Frank, 2016; Demetrioff et al., 2017; Felisberti, 2018; Zeng et al., 2018; Zhang et al., 2020a).^4^ In addition, given that previous studies indicate that the duration of micro-expressions can significantly affect the recognition accuracy of micro-expressions (e.g., Matsumoto et al., 2000; Shen et al., 2012; Wu et al., 2016; Zeng et al., 2018; Xie et al., 2019), we employed three different settings of duration (i.e., 50, 150, and 333 ms) to test our hypothesis in Study 1.

### Method

#### Participants and Design

The G*Power software (Faul et al., 2009) was used to estimate the required sample size. By using the average effect size of oxytocin on social behaviors (*f* = 0.14; Walum et al., 2016) as our effect size estimation and giving the current experimental design, the sample size calculation estimated a sample of 72 to achieve the power of 0.9 (α = 0.05). Finally, we recruited 72 Chinese adult male participants (*M*_age_ = 19.99, *SD* = 1.67; oxytocin condition: *n* = 36; placebo condition: *n* = 36) through advertisements on campus. All participants were healthy and had normal or corrected-to-normal vision and no history of neurological or psychiatric disorders. All participants had not taken any medications within 1 month before the experiment and all participants reported that they did not have any history of allergic reactions. Moreover, all participants were asked to abstain from smoking, doing sports, drinking coffee or alcohol in the 24 h before the experiment, and from eating or drinking (other than water) for 2 h before the experiment. All participants self-identified as heterosexual. This study was approved by the Research Ethics Committee of Hunan Normal University (No. 2019.192). Written informed consent in accordance with the Declaration of Helsinki was obtained from all participants. None of the participants had participated in other studies of the present research. All participants were paid for their participation.

A 2 (treatment: oxytocin, placebo) × 6 (emotion category: sadness, surprise, anger, disgust, fear, and happiness) × 3 (duration: 50, 150, and 333 ms) mixed-model experimental design was used, with treatment being the between-subjects factor while emotion category and duration being the within-subjects factors.

#### Standardized Micro-Expression Recognition Task

Thirty-six models were randomly selected from the BU-3DFE database (Yin et al., 2006). This database contains neutral and six universal facial expressions (sadness, surprise, anger, disgust, fear, and happiness; with four different intensity levels in facial actions, including low, middle, high, and very high) from 100 models (56 females and 44 males, aged 18–70 years, with a variety of ethnic backgrounds, including White, Black, East-Asian, Middle-Asian, Indian, and Hispanic). For these selected models, the images of their neutral faces and the images of their six basic facial expressions (i.e., sadness, surprise, anger, disgust, fear, and happiness) that were rated to be “very high” in the intensity of facial actions (Yin et al., 2006; Wu et al., 2016; Zeng et al., 2018) were selected. Therefore, a total of 252 images were selected as the stimuli of Study 1. These selected models were then randomly divided into three sets (i.e., 12 models in each set) and these three sets were randomly assigned to the three different duration conditions. All of the six categories of micro-expressions of the models were presented according to their assigned duration condition (50, 150, or 333 ms).

**Figure 1:**
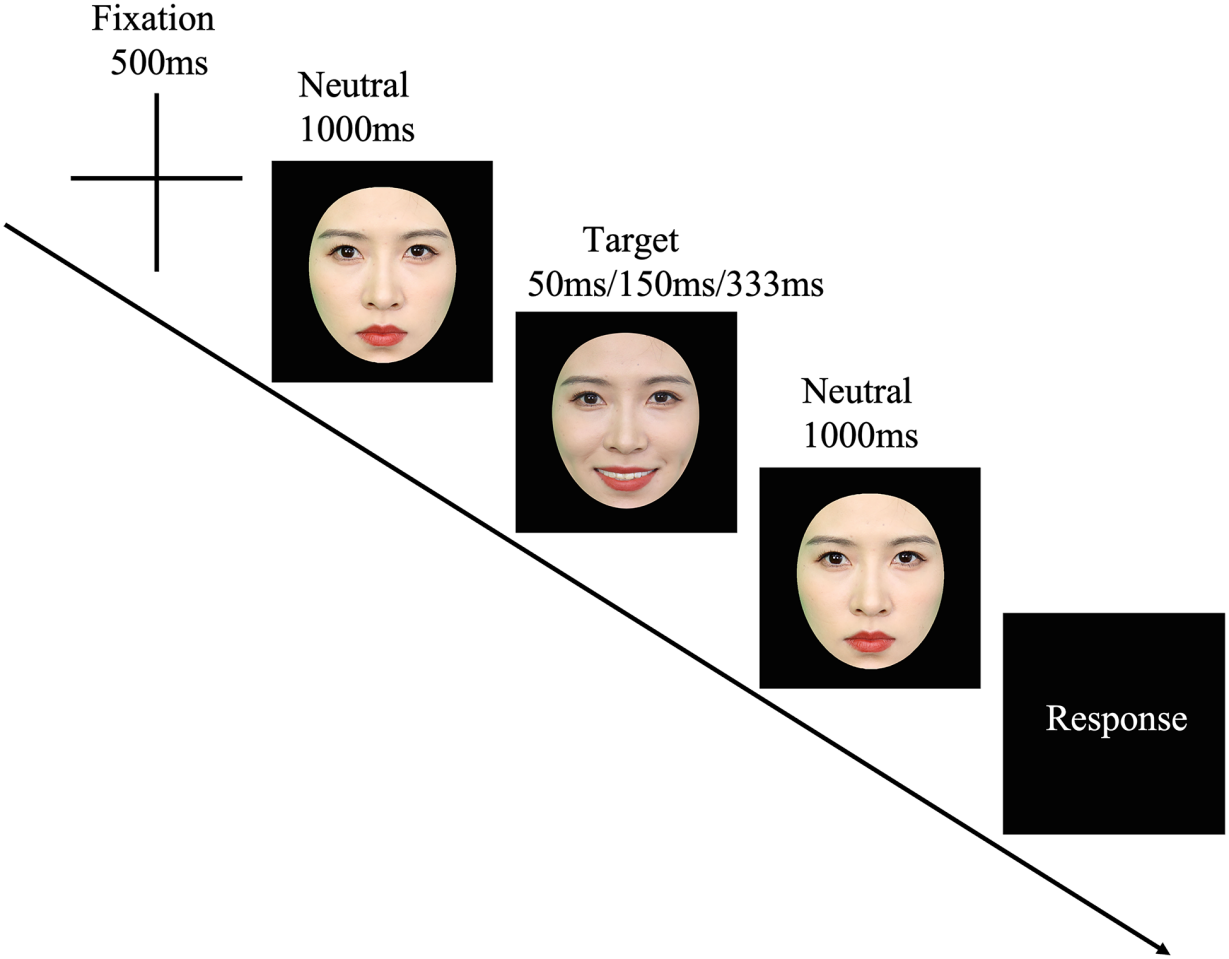
The JACBART paradigm. Note that we use the facial images of the second author for illustration.

The JACBART paradigm was employed to present the micro-expressions (Matsumoto et al., 2000; Matsumoto and Hwang, 2011; Shen et al., 2012; Svetieva and Frank, 2016; Zeng et al., 2018). Firstly, a fixation cross (500 ms) was presented at the center of the screen, then the facial expression image (presented for 50, 150, or 333 ms) was sandwiched in between two 1 s presentations of the same expresser’s neutral face (see Figure 1). After that, participants were asked to choose one of seven emotion labels (i.e., sadness, happiness, fear, surprise, anger, disgust, and a label of “none of these emotions”) from a list to describe the expression just displayed by using the mouse. They were asked to respond as accurately and as fast as possible. The presentation order of the stimulus and the order of the seven emotion labels in the list were completely randomized. The order of the combination of the model sets and the duration conditions was counterbalanced across participants, and each micro-expression of each model was presented only once. Therefore, there were 216 trials in total. These trials were further divided into three blocks (each block had 72 trials), and a two-minute break was put between blocks. For the standardized micro-expression recognition task, the recognition accuracy and reaction time (RT) were recorded.

#### Procedure

Upon arrival, participants first completed the Positive and Negative Affect Schedule (PANAS; Watson et al., 1988) to assess their current mood. The PANAS is a self-report questionnaire that consists of two 10-item scales to measure both positive (before treatment: Cronbach α = 0.82) and negative affect (before treatment: Cronbach α = 0.84). Higher scores on these measures indicate greater positive or negative affect.

After finishing the PANAS, participants were randomly assigned to the oxytocin or placebo condition. These two treatments were administrated in a double-blind fashion. In accordance with a standardized protocol and under experimenter supervision, the participants self-administered five puffs of oxytocin (Oxytocin Nasal Spray, Moike, China; 4 IU oxytocin per puff, total dose of 40 IU oxytocin) or placebo (containing all ingredients except for the neuropeptide) per nostril (e.g., Leknes et al., 2012; Shin et al., 2018; for review, see Romero-Martínez et al., 2021). After a 45 min loading period, during which the participants watched a neutral movie with non-social content (Leknes et al., 2012; Shin et al., 2018; Schwaiger et al., 2019), the PANAS was completed again to assess the positive (after treatment: Cronbach α = 0.89) and negative affect (after treatment: Cronbach α = 0.88) of participants for the second time. Then all participants were asked to finish the standardized micro-expression recognition task.

### Results and Discussion

Rating scores for the positive and negative affect were subjected to a 2 (treatment: oxytocin, placebo) × 2 (time: before, after) mixed-model analysis of variance (ANOVA). The results showed that the main effect of time was significant for both positive and negative affect (*F*s > 75.83, *p*s < 0.001), participants displayed less positive (before: *M* = 32.39, *SD* = 6.26; after: *M* = 22.5, *SD* = 6.73) and negative (before: *M* = 30.14, *SD* = 7.68; after: *M* = 19.5, *SD* = 6.98) affect after receiving the treatment. But the main effect of treatment and the interaction between treatment and time were not significant for both positive and negative affect (*F*s < 3.03, *p*s > 0.08). These results indicate that the administration of intranasal oxytocin did not differentially affect the mood of participants and therefore they rule out the possibility that the effects of oxytocin were caused by its differential effect on participants’ mood.

The accuracy data of standardized micro-expression recognition task were then subjected to a 2 (treatment) × 6 (emotion category) × 3 (duration) mixed-model ANOVA. The results showed that the main effect of treatment [*F* (1, 70) = 1, *p* = 0.32, η_p_^2^ = 0.01], and the effects of treatment × emotion category [*F* (5, 350) = 0.89, *p* = 0.49, η_p_^2^ = 0.01], treatment × duration [*F* (2, 140) = 0.46, *p* = 0.63, η_p_^2^ = 0.01], and treatment × emotion category × duration [*F* (10, 700) = 1.2, *p* = 0.29, η_p_^2^ = 0.02] were all not significant. These results indicate that intranasal oxytocin did not significantly affect the recognition accuracy of standardized intense micro-expressions (see Table 1).

**Table 1:** The recognition accuracy of standardized micro-expression recognition task in Study 1 (*M* ± *SD*).

	50 ms		150 ms		333 ms	
	Oxytocin	Placebo	Oxytocin	Placebo	Oxytocin	Placebo
Sadness	0.23 ± 0.18	0.22 ± 0.14	0.42 ± 0.18	0.44 ± 0.21	0.51 ± 0.21	0.48 ± 0.23
Surprise	0.7 ± 0.21	0.72 ± 0.23	0.75 ± 0.18	0.78 ± 0.24	0.75 ± 0.21	0.77 ± 0.19
Anger	0.07 ± 0.09	0.15 ± 0.16	0.11 ± 0.12	0.19 ± 0.13	0.12 ± 0.15	0.15 ± 0.11
Disgust	0.21 ± 0.13	0.20 ± 0.16	0.41 ± 0.19	0.34 ± 0.20	0.44 ± 0.15	0.46 ± 0.22
Fear	0.1 ± 0.12	0.09 ± 0.09	0.07 ± 0.08	0.11 ± 0.12	0.17 ± 0.14	0.18 ± 0.12
Happiness	0.68 ± 0.21	0.76 ± 0.25	0.85 ± 0.15	0.86 ± 0.12	0.86 ± 0.11	0.87 ± 0.16

The RT data of micro-expression recognition task (log transformed prior to analysis) were also subjected to a 2 (treatment) × 6 (emotion category) × 3 (duration) mixed-model ANOVA. The results revealed a significant treatment × emotion category × duration interaction [*F* (10, 700) = 1.0, *p* = 0.04, η_p_^2^ = 0.04], whereas the main effect of treatment [*F* (1, 70) = 0.09, *p* = 0.76, η_p_^2^ = 0.001] and the effects of treatment × emotion category [*F* (5, 350) = 1.41, *p* = 0.22, η_p_^2^ = 0.02] and treatment × duration [*F* (2, 140) = 1, *p* = 0.37, η_p_^2^ = 0.01] were not significant. Further simple effects analysis showed that intranasal oxytocin significantly increased the RTs for the recognition of surprised micro-expressions under the condition of 150 ms, *F* (1, 70) = 4.82, *p* = 0.03, η_p_^2^ = 0.06. There was also a trend for participants to increase the RTs for the recognition of fearful micro-expressions under the condition of 50 ms, *F* (1, 70) = 3.38, *p* = 0.07, η_p_^2^ = 0.05, but the other simple effects were not significant, *F*s < 1.05, *p*s > 0.3 (see Table 2).

**Table 2:** The RT (log transformed) of standardized micro-expression recognition task in Study 1 (*M* ± *SD*).

	50 ms		150 ms		333 ms	
	Oxytocin	Placebo	Oxytocin	Placebo	Oxytocin	Placebo
Sadness	3.33 ± 0.17	3.36 ± 0.15	3.37 ± 0.15	3.36 ± 0.13	3.36 ± 0.12	3.36 ± 0.12
Surprise	3.31 ± 0.14	3.29 ± 0.11	3.34 ± 0.16	3.27 ± 0.11	3.29 ± 0.12	3.27 ± 0.11
Anger	3.37 ± 0.18	3.34 ± 0.18	3.39 ± 0.15	3.37 ± 0.14	3.4 ± 0.16	3.43 ± 0.12
Disgust	3.39 ± 0.18	3.36 ± 0.14	3.38 ± 0.14	3.37 ± 0.12	3.39 ± 0.14	3.37 ± 0.12
Fear	3.38 ± 0.17	3.31 ± 0.14	3.33 ± 0.16	3.36 ± 0.11	3.37 ± 0.15	3.37 ± 0.11
Happiness	3.25 ± 0.16	3.26 ± 0.16	3.23 ± 0.16	3.25 ± 0.14	3.22 ± 0.12	3.24 ± 0.15

In sum, in Study 1 we found that a single dose of intranasally administrated oxytocin could significantly decrease the recognition speed for standardized intense micro-expressions of surprise under the 150 ms duration condition. This result is consistent with our hypothesis which suggests that oxytocin may have an emotion-specific inhibiting role in recognition of intense micro-expression of surprise. We also found a trend for participants to decrease their recognition speed for standardized intense micro-expressions of fear under the 50 ms condition in Study 1. However, it should be noted that the acquired sample size of Study 1 was completely consistent with our estimated sample size, which means that we should have enough power (no less than 0.9) to detect small-to-medium interaction effects in Study 1. Therefore, such a trend should be better to be treated as nonsignificant and be interpreted with caution.

## Study 2

Study 1 only investigated the effects of oxytocin on standardized intense micro-expressions. However, researchers have found that usually the intensity of micro-expressions is low (Porter and ten Brinke, 2008; Yan et al., 2013) and the recognition process of standardized subtle micro-expressions may be significantly different from the recognition process of standardized intense micro-expressions (Wu et al., 2016; Zeng et al., 2018). Therefore, we further tested the effects of intranasal oxytocin on the recognition of standardized subtle micro-expressions in Study 2.

### Method

#### Participants and Design

G*Power software was used to acquire *a priori* estimate of the required sample size. By using the same parameters of Study 1 (power = 0.9, effect size *f* = 0.14, α = 0.05) and giving the current experimental design, the analysis estimated a sample size of 72. We finally recruited 75 Chinese adult male participants (*M*_age_ = 19.06, *SD* = 1.32; oxytocin condition: *n* = 39; placebo condition: *n* = 36) through advertisements on campus. The requirements for participants were completely consistent with Study 1 (i.e., they had to be healthy and had normal or corrected-to-normal vision, no history of neurological or psychiatric disorders, etc.). This study was approved by the Research Ethics Committee of Hunan Normal University (No. 2019.192). Written informed consent in accordance with the Declaration of Helsinki was obtained from all participants. None of the participants had participated in other studies of the present research. All participants were paid for their participation.

A 2 (treatment: oxytocin, placebo) × 6 (emotion category: sadness, surprise, anger, disgust, fear, and happiness) × 3 (duration: 50, 150, and 333 ms) mixed-model experimental design was used, with treatment being the between-subjects factor while emotion category and duration being the within-subjects factors.

#### Materials and Procedure

Similar to Study 1, participants were also asked the finish the PANAS to assess their current positive (before treatment: Cronbach α = 0.82) and negative affect (before treatment: Cronbach α = 0.8) at first. Then participants received a single dose (40 IU) of intranasally administrated oxytocin or placebo in the way that was completely consistent with Study 1. After that, participants were also asked to wait for 45 min during which they were instructed to watch a neutral movie with non-social content as in Study 1. Then the PANAS was completed again to assess the positive (after treatment: Cronbach α = 0.89) and negative affect (after treatment: Cronbach α = 0.87) of participants. Lastly, participants were asked to finish a standardized micro-expression recognition task that was identical to Study 1 except that the facial stimuli of this task were very low in the intensity level (i.e., facial expressions of the 36 models of BU-3DFE database that were rated to be “low” in the intensity level were employed).

### Results and Discussion

As in Study 1, the rating scores of PANAS were subjected to 2 (treatment) × 2 (time) mixed-model ANOVAs. The results showed that the main effect of treatment and the treatment × time interaction were not significant for both positive and negative affect (*F*s < 1.1, *p*s > 0.3), but the main effect of time was significant for both the positive and the negative affect (*F*s > 28.55, *p*s < 0.001). That is, similar to Study 1, participants displayed less positive (before: *M* = 31.65, *SD* = 6.02; after: *M* = 26.56, *SD* = 7.8) and less negative (before: *M* = 29.91, *SD* = 7.26; after: *M* = 18.39, *SD* = 6.3) affect after receiving the treatment.

**Figure 2:**
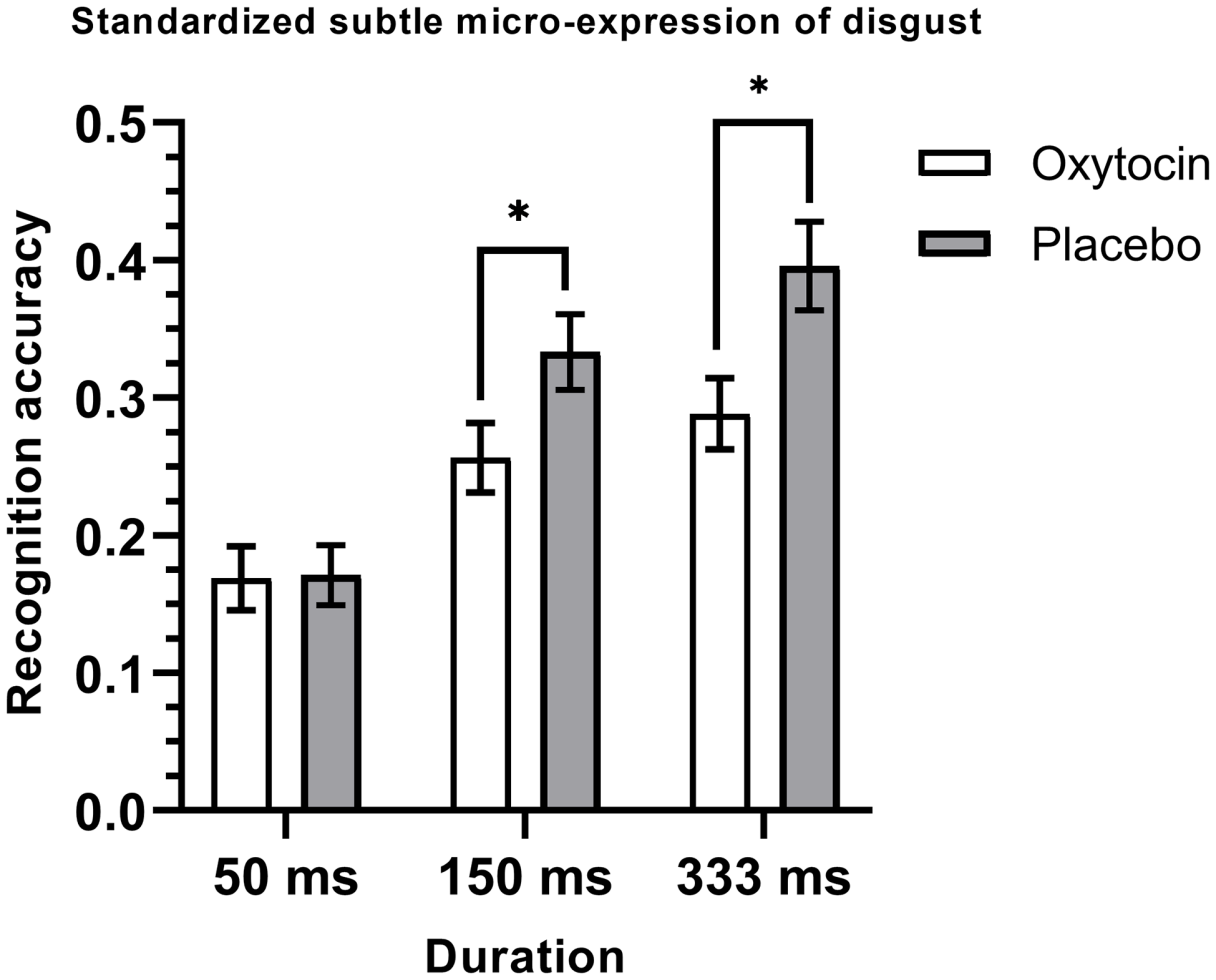
Mean recognition accuracies of standardized subtle micro-expressions of disgust. Error bars represent standard errors. The symbol * indicates that the differences were significant.

The 2 (treatment) × 6 (emotion category) × 3 (duration) mixed-model ANOVA on the recognition accuracy of standardized micro-expression recognition task showed that the treatment × emotion category × duration interaction was significant, *F* (10, 730) = 1.9, *p* = 0.04, η_p_^2^ = 0.03, but the main effect of treatment [*F* (1, 73) = 2.41, *p* = 0.13, η_p_^2^ = 0.03], and the effects of treatment × emotion category [*F* (5, 365) = 0.95, *p* = 0.45, η_p_^2^ = 0.01] and treatment × duration [*F* (2, 146) = 0.01, *p* = 0.99, η_p_^2^ < 0.001] were not significant. Further simple effects analysis showed that intranasal oxytocin significantly decreased the recognition accuracy for the micro-expression of disgust. More specifically, this effect was significant under the duration conditions of 150 ms [*F* (1, 73) = 4.24, *p* = 0.04, η_p_^2^ = 0.06] and 333 ms [*F* (1, 73) = 6.87, *p* = 0.01, η_p_^2^ = 0.09], but it was not significant under the 50 ms condition [*F* (1, 73) = 0.01, *p* = 0.94, η_p_^2^ < 0.001] (see Figure 2). The other simple effects were all not significant, *F*s < 1.77, *p*s > 0.18 (see Table 3).

**Table 3:** The recognition accuracy of standardized micro-expression recognition task in Study 2 (*M* ± *SD*).

	50 ms		150 ms		333 ms	
	Oxytocin	Placebo	Oxytocin	Placebo	Oxytocin	Placebo
Sadness	0.11 ± 0.11	0.13 ± 0.09	0.26 ± 0.17	0.29 ± 0.16	0.33 ± 0.2	0.35 ± 0.2
Surprise	0.45 ± 0.21	0.52 ± 0.24	0.63 ± 0.24	0.68 ± 0.2	0.71 ± 0.24	0.74 ± 0.16
Anger	0.08 ± 0.12	0.09 ± 0.11	0.14 ± 0.12	0.11 ± 0.12	0.12 ± 0.11	0.15 ± 0.15
Disgust	0.17 ± 0.14	0.17 ± 0.13	0.26 ± 0.16	0.33 ± 0.17	0.29 ± 0.16	0.4 ± 0.19
Fear	0.08 ± 0.09	0.06 ± 0.07	0.08 ± 0.07	0.08 ± 0.09	0.1 ± 0.08	0.09 ± 0.1
Happiness	0.5 ± 0.24	0.56 ± 0.25	0.68 ± 0.22	0.69 ± 0.18	0.79 ± 0.17	0.74 ± 0.17

The 2 (treatment) × 6 (emotion category) × 3 (duration) mixed-model ANOVA on the RT data of standardized micro-expression recognition task (log transformed prior to analysis) revealed that the main effect of treatment [*F* (1, 73) = 0.15, *p* = 0.7, η_p_^2^ = 0.002], and the effects of treatment × emotion category [*F* (5, 365) = 0.91, *p* = 0.47, η_p_^2^ = 0.01], treatment × duration [*F* (2, 146) = 0.12, *p* = 0.89, η_p_^2^ = 0.01], and treatment × emotion category × duration [*F* (10, 730) = 1.23, *p* = 0.27, η_p_^2^ = 0.02] were all not significant (see Table 4). These results indicate that intranasal oxytocin did not affect the recognition speed of subtle micro-expressions.

**Table 4:** The RT (log transformed) of standardized micro-expression recognition task in Study 2 (*M* ± *SD*).

	50 ms		150 ms		333 ms	
	Oxytocin	Placebo	Oxytocin	Placebo	Oxytocin	Placebo
Sadness	3.32 ± 0.18	3.33 ± 0.2	3.35 ± 0.16	3.37 ± 0.17	3.37 ± 0.15	3.34 ± 0.15
Surprise	3.35 ± 0.19	3.32 ± 0.19	3.3 ± 0.15	3.31 ± 0.17	3.29 ± 0.14	3.3 ± 0.14
Anger	3.3 ± 0.19	3.32 ± 0.2	3.35 ± 0.15	3.39 ± 0.18	3.33 ± 0.15	3.38 ± 0.15
Disgust	3.34 ± 0.15	3.34 ± 0.17	3.34 ± 0.15	3.34 ± 0.17	3.34 ± 0.14	3.39 ± 0.16
Fear	3.29 ± 0.17	3.34 ± 0.16	3.33 ± 0.13	3.32 ± 0.16	3.33 ± 0.15	3.33 ± 0.17
Happiness	3.28 ± 0.14	3.28 ± 0.17	3.26 ± 0.13	3.27 ± 0.18	3.25 ± 0.12	3.26 ± 0.14

In summary, consistent with Study 1, the results of Study 2 showed an emotion-specific inhibiting effect of intranasal oxytocin on the recognition of micro-expressions. Specifically, the results showed that a single dose of intranasal oxytocin could significantly decrease the recognition accuracy for the standardized subtle micro-expressions of disgust under the duration conditions of 150 ms and 333 ms. These results were consistent with our hypothesis which suggests that oxytocin may have an emotion-specific inhibiting role in the recognition of subtle micro-expression of disgust. It should be noted that the inhibiting effect of oxytocin on the recognition of subtle micro-expression of disgust was not significant under the 50 ms condition, which was probably caused by the floor effect (i.e., it was very difficult to recognize the disgusted micro-expressions under this condition, the accuracy was only about 0.17).

## Study 3

In Study 1 and Study 2, we employed the JACBART paradigm to present standardized micro-expressions. Compared with presenting natural micro-expressions (i.e., micro-expressions that are elicited under real social situations), utilizing standardized micro-expressions has the advantage that it can effectively control the feature of micro-expressions (e.g., the duration and intensity of micro-expressions) and minimize the interference of irrelevant factors (e.g., lighting condition of facial images, head movement and eye blinks of the models, irrelevant facial actions caused by speech; Matsumoto et al., 2000; Ekman, 2009; Matsumoto and Hwang, 2011; Hurley et al., 2014; Svetieva and Frank, 2016; Demetrioff et al., 2017; Felisberti, 2018; Zeng et al., 2018). However, since the JACBART paradigm only uses three sequential facial images to present micro-expressions, the facial dynamics of standardized micro-expressions is significantly different from the facial dynamics of natural micro-expressions (Yan et al., 2013; Svetieva and Frank, 2016; Zeng et al., 2018). Previous researches also suggest that the dynamical information of facial actions is crucial in the processing of facial expressions (e.g., Kamachi et al., 2013; Sato et al., 2014). Therefore, to further test our hypothesis, we investigated the effects of intranasal oxytocin on the recognition of natural micro-expressions in Study 3. Given that the results of Study 1 and Study 2 suggest that oxytocin may have an inhibiting role in the recognition of standardized surprised and disgusted micro-expressions, we predicted that a single dose of intranasally administrated oxytocin would also inhibit the recognition of natural micro-expressions of surprise and disgust.

Previous studies have found that usually the recognition accuracy for natural micro-expressions was very low (mean accuracy was only about 0.2; Frank et al., 2009; Svetieva and Frank, 2016). However, previous studies also suggest that the recognition accuracy of micro-expressions could be significantly improved after receiving trainings in micro-expression recognition (e.g., Frank et al., 2009; Matsumoto and Hwang, 2011; Hurley et al., 2014). To avoid the potential floor effect, a validated self-instructional training package of Micro-Expression Training Tool (METT; Ekman, 2002, 2009; Russell et al., 2008; Endres and Laidlaw, 2009; Frank et al., 2009; Weinberger, 2010; Matsumoto and Hwang, 2011; Hurley et al., 2014; Frank and Svetieva, 2015) was employed (in the training condition) to improve the micro-expression recognition ability of participants in Study 3.

### Method

#### Participants and Design

G*Power software was used to acquire *a priori* estimate of the required sample size. By using the same parameters of Study 1 (power = 0.9, effect size *f* = 0.14, α = 0.05) and giving the current experimental design, the analysis estimated a sample size of 144. Due to the constraints of available time and available participants at the end of an academic semester, we finally recruited 126 Chinese adult male participants (*M*_age_ = 21.37, *SD* = 2.84; training condition: *n* = 56; no training condition: *n* = 70) through advertisements on campus. The requirements for participants were completely consistent with Study 1. Sensitivity power analysis indicated that the minimal detectable effect (power = 0.8) of the acquired sample size was *f* = 0.14 for the training condition and *f* = 0.12 for the no training condition. This study was approved by the Research Ethics Committee of Hunan Normal University (No. 2019.192). Written informed consent in accordance with the Declaration of Helsinki was obtained from all participants. None of the participants had participated in other studies of the present research. All participants were paid for their participation.

A 2 (group: training, no training) × 2 (treatment: oxytocin, placebo) × 6 (emotion category: sadness, surprise, anger, disgust, fear, and happiness) mixed-model experimental design was used, with emotion category being the within-subjects factor while group and treatment being the between-subjects factors.

#### Natural Micro-Expression Recognition Task

Natural micro-expression samples were selected from two of the currently largest natural micro-expression databases (i.e., the CASME II database and the SAMM Long Videos database; Yan et al., 2014; Yap et al., 2020). In these two databases, natural micro-expressions were elicited by a neutral face paradigm in which the models were asked to keep a neutral face while watching videos with strong emotional contents. The elicited micro-expressions were recorded by high-speed cameras which ran at a frame rate of 200 fps. In Study 3, 94 micro-expression samples with clear emotion labels were selected from these two databases. Specifically, 9 micro-expressions of sadness, 19 micro-expressions of surprise, 18 micro-expressions of anger, 21 micro-expressions of disgust, 7 micro-expressions of fear, and 20 micro-expressions of happiness were selected.^5^ For these selected samples, we only imported the video frames which contained the micro-expressions and the 100 video frames which contained the neutral faces of the models before and after the video frames of micro-expressions. Then, these imported video frames were converted to gray-scale and these edited micro-expression samples were employed as the stimuli in Study 3.

In the natural micro-expression recognition task of Study 3, a fixation cross (500 ms) was presented at the center of the screen, then the natural micro-expression video was displayed at the same location. After that, participants were asked to choose one of eight emotion labels (i.e., sadness, happiness, fear, surprise, anger, disgust, neutral emotion, and a label of “none of these emotions”) from a list to describe the expression just displayed by using the mouse (Svetieva and Frank, 2016), and they were asked to respond as accurately and as fast as possible. The presentation order of the stimulus and the order of the eight emotion labels in the list were completely randomized. Each micro-expression was presented only once. Therefore, there were 94 trials in total. These trials were divided into two blocks (each block had 47 trials), and a 30 s break was put between the two blocks. The recognition accuracy and RT were recorded in this task.

#### Micro-Expression Recognition Training

METT is a self-instructional micro-expression recognition training program (Ekman, 2002). This training program has been shown to be able to improve the recognition accuracy of standardized and natural micro-expressions for participants from various backgrounds (e.g., university students, school teachers, business persons, Coast Guard personnel, etc.; Ekman, 2002, 2009; Russell et al., 2008; Endres and Laidlaw, 2009; Frank et al., 2009; Weinberger, 2010; Matsumoto and Hwang, 2011; Hurley et al., 2014; Frank and Svetieva, 2015). Therefore, we employed the METT to perform micro-expression recognition training in Study 3. Specifically, participants were asked to complete the pre- and post-test, training, practice and review sections of METT under the supervision of experimenter, which took about 30 min.

In the pre-test, participants viewed 14 standardized micro-expressions (presented by employing the JACBART paradigm), consisting of either, disgust, sadness, happiness, contempt, fear, anger or surprise. Participants were prompted to select one of the seven emotional labels to describe the micro-expression just displayed. The mean accuracy was displayed and recorded after the completion of pre-test. Then participants were asked to finish the training, the practice, and the review section of METT. In the training and review sessions, participants were asked to watch videos and learn the skills about how to distinguish between commonly confused emotions (e.g., surprise/fear, fear/sadness). In the practice session, participants were instructed to practice with 28 standardized micro-expressions identical to the pre-test, supplemented with feedback after each selection of micro-expression displayed. After the review, participants were asked to finish the post-test which had the same procedure as the pre-test. The mean accuracy was also displayed and recorded.

#### Procedure

Due to limitations in the research resources available, Study 3 was first conducted under the no training condition, then the training condition was carried out. Participants in the no training condition did not receive any trainings in micro-expression recognition a day before the formal experiment, whereas the participants in the training condition were asked to finish the training of METT a day before the formal experiment.

In the formal experiment, similar to Study 1 and Study 2, participants were instructed to finish the PANAS at first (positive affect before treatment: Cronbach α = 0.82; negative affect before treatment: Cronbach α = 0.84). Then, the intranasal oxytocin or placebo was administrated in the way that was completely consistent with Study 1. After that, participants were also asked to wait for 45 min during which they were instructed to watch a neutral movie with non-social content as in Study 1. Then the PANAS was completed again (positive affect after treatment: Cronbach α = 0.89; negative affect after treatment: Cronbach α = 0.88). Lastly, participants were asked to finish the natural micro-expression recognition task.

### Results and Discussion

The 2 (treatment) × 2 (time) mixed-model ANOVAs on the PANAS data showed that for both the training and no training conditions, participants displayed less negative affect after treatment, *F*s > 15.52, *p*s < 0.001, but their positive affect was not affected, *F*s < 1.32, *p*s > 0.25 (see Table 5). Furthermore, the main effect of treatment and the interaction of treatment and time were all not significant for both the training and no training conditions, *F*s < 0.64, *p*s > 0.42. Therefore, these results indicate that the intranasal oxytocin administration also did not differentially affect participants’ mood in Study 3. Further analysis also showed that there were no significant differences in the positive and negative affect among the different experimental conditions (i.e., training-oxytocin, training-placebo, no training-oxytocin, no training-placebo) before (*F*s < 0.2, *p*s > 0.89) or after (*F*s < 0.82, *p*s > 0.48) the treatment.

**Table 5:** The positive and negative affect of participants in Study 3 (*M* ± *SD*).

	Training		No training	
	Oxytocin	Placebo	Oxytocin	Placebo
Positive-before	32.64 ± 3.09	32.25 ± 3.08	32.97 ± 4.52	32.46 ± 6.46
Positive-after	32.54 ± 3.21	31.71 ± 2.67	32.66 ± 5.51	32.06 ± 6.72
Negative-before	21.89 ± 3.49	22.32 ± 3.2	22.69 ± 5.45	21.94 ± 5.64
Negative-after	20.64 ± 3.34	20.64 ± 3.76	19.74 ± 6.06	18.89 ± 6.45

Subsequent manipulation checks on the training effect of micro-expression recognition showed that the training of METT was effective, participants in the training condition displayed higher recognition accuracy [*F* (1, 54) = 397.41, *p* < 0.001, η_p_^2^ = 0.88] for standardized micro-expressions after the training of METT (pre-test: *M* = 36.52, *SD* = 12.91; post-test: *M* = 63.54. *SD* = 12.68). The results also showed that the participants in the training condition (*M* = 0.27. *SD* = 0.05) displayed higher recognition accuracy for natural micro-expressions than the participants of the no training condition (*M* = 0.22. *SD* = 0.06), *t* (124) = 5.14, *p* < 0.001, Cohen’s *d* = 0.92, which indicate that the training of METT was also effective for natural micro-expressions. Further analysis on the RT data of natural micro-expression task (log transformed prior to analysis) showed that the METT training did not affect the recognition speed of natural micro-expressions, *t* (110.24) = 0.96, *p* = 0.34, Cohen’s *d* = 0.17.

**Figure 3:**
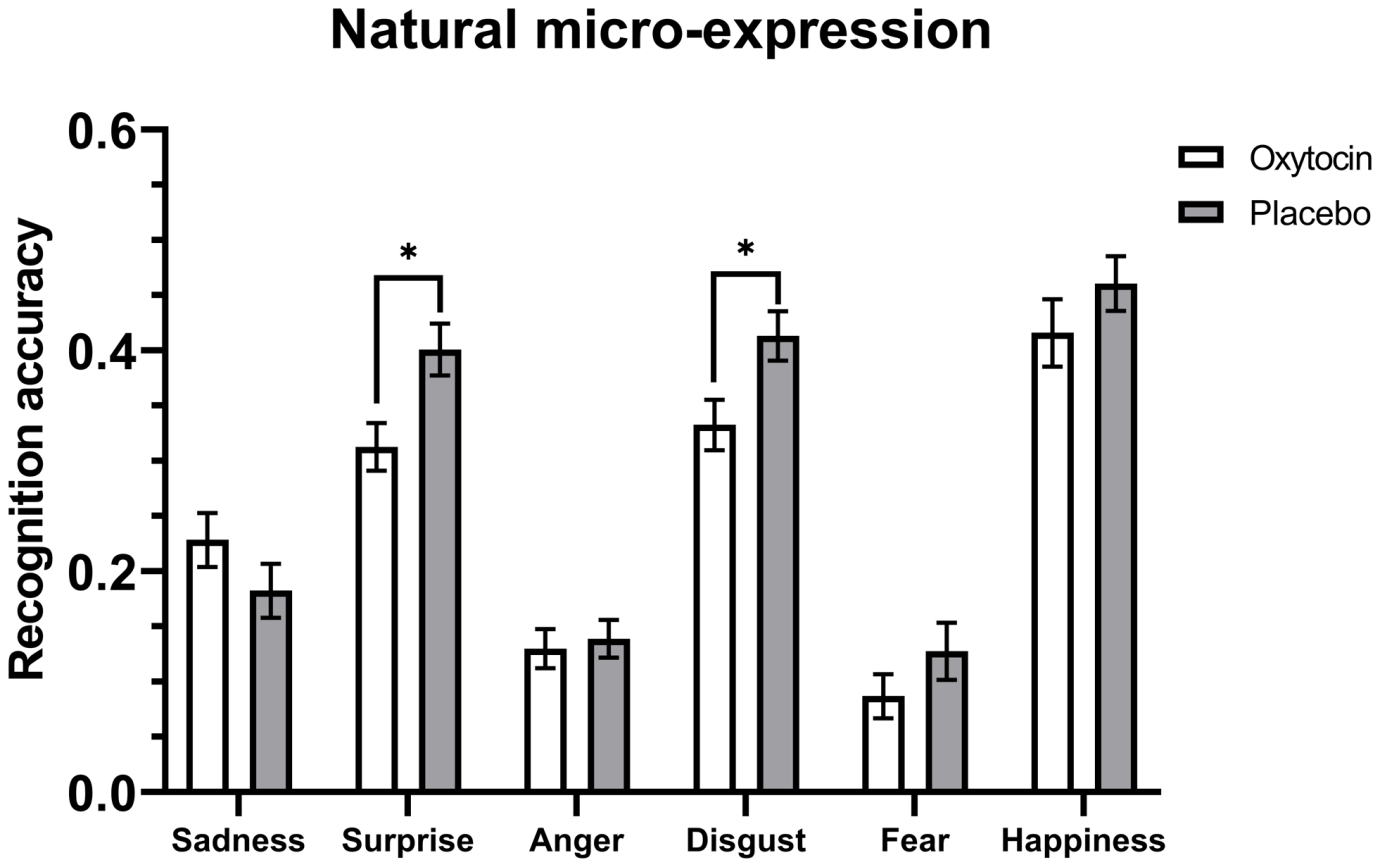
Mean recognition accuracies of natural micro-expressions under the training condition. Error bars represent standard errors. The symbol * indicates that the differences were significant.

Further 2 (treatment) × 6 (emotion category) mixed-model ANOVAs on the accuracy data of natural micro-expression recognition task showed that for the training condition, the main effect of treatment [*F* (1, 54) = 6.99, *p* = 0.01, η_p_^2^ = 0.12] and the interaction between treatment and emotion category [*F* (5, 270) = 2.31, *p* = 0.045, η_p_^2^ = 0.04] were significant, but these effects were not significant for the no training condition, *F*s < 0.75, *p*s > 0.58 (see Table 6). Further simple effects analysis showed that under the training condition, intranasal oxytocin decreased the recognition accuracy for natural micro-expressions of surprise (*F* (1, 54) = 7.64, *p* = 0.008, η_p_^2^ = 0.12) and disgust (*F* (1, 54) = 6.4, *p* = 0.01, η_p_^2^ = 0.11), but it did not affect the recognition accuracy for other natural micro-expressions, *F*s < 1.78, *p*s > 0.18 (see Figure 3).

**Table 6:** The recognition accuracy of natural micro-expression recognition task in Study 3 (*M* ± *SD*).

	Training		No training	
	Oxytocin	Placebo	Oxytocin	Placebo
Sadness	0.23 ± 0.13	0.18 ± 0.13	0.11 ± 0.13	0.15 ± 0.17
Surprise	0.31 ± 0.11	0.4 ± 0.12	0.28 ± 0.14	0.31 ± 0.13
Anger	0.13 ± 0.09	0.14 ± 0.09	0.08 ± 0.08	0.07 ± 0.07
Disgust	0.33 ± 0.12	0.41 ± 0.12	0.26 ± 0.17	0.25 ± 0.11
Fear	0.09 ± 0.11	0.13 ± 0.14	0.11 ± 0.13	0.11 ± 0.12
Happiness	0.42 ± 0.16	0.46 ± 0.13	0.46 ± 0.12	0.42 ± 0.18

The 2 (treatment) × 6 (emotion category) mixed-model ANOVAs on the RT data of natural micro-expression recognition task (log transformed prior to analysis) revealed that the main effect of treatment and the effect of treatment × emotion category were all not significant for both training and no training conditions, *F*s < 3.05*, p*s > 0.08 (see Table 7). These results indicate that intranasal oxytocin did not affect the recognition speed for natural micro-expressions.

**Table 7:** The RT (log transformed) of natural micro-expression recognition task in Study 3 (M ± SD).

	Training		No training	
	Oxytocin	Placebo	Oxytocin	Placebo
Sadness	3.54 ± 0.13	3.6 ± 0.13	3.52 ± 0.12	3.53 ± 0.19
Surprise	3.54 ± 0.1	3.6 ± 0.12	3.52 ± 0.11	3.52 ± 0.12
Anger	3.58 ± 0.13	3.56 ± 0.14	3.57 ± 0.16	3.54 ± 0.15
Disgust	3.5 ± 0.08	3.58 ± 0.1	3.54 ± 0.13	3.54 ± 0.12
Fear	3.56 ± 0.11	3.53 ± 0.11	3.53 ± 0.19	3.54 ± 0.18
Happiness	3.46 ± 0.15	3.48 ± 0.16	3.51 ± 0.09	3.51 ± 0.13

Consistent with previous researches (Ekman, 2002, 2009; Russell et al., 2008; Endres and Laidlaw, 2009; Frank et al., 2009; Weinberger, 2010; Matsumoto and Hwang, 2011; Hurley et al., 2014; Frank and Svetieva, 2015), in Study 3 we found that the METT training significantly improved participants’ ability to read micro-expressions. The results also showed that intranasal oxytocin significantly decreased the recognition accuracy for natural micro-expressions of surprise and disgust after the training of METT, but it did not affect the recognition speed for all the natural micro-expressions. These results were consistent with our hypothesis, they suggest that the oxytocin system has participated in the processing of micro-expressions but it mainly plays an inhibiting role, especially for the recognition of micro-expressions of surprise and disgust.

## General Discussion

The neuropeptide oxytocin has attracted immerse research attention for its central role in the expression of the high levels of sociality that are essential to contemporary human behavior (Carter, 2014; Ma et al., 2016; Leppanen et al., 2017; Xu et al., 2019; Quintana et al., 2021; Romero-Martínez et al., 2021). By employing intranasal administration of oxytocin, the present work is the first to demonstrate that oxytocin may have an emotion-specific inhibiting effect on the recognition of micro-expressions of six basic emotions. Specifically, in Study 1, we found that intranasal oxytocin decreased the recognition speed for the standardized intense micro-expression of surprise under the duration condition of 150 ms. In Study 2, we found that intranasal oxytocin also decreased the recognition accuracy for the standardized subtle micro-expression of disgust under the duration conditions of 150 and 333 ms. In Study 3, we further investigated the effects of intranasal oxytocin on the recognition of natural micro-expressions, and the results consistently showed that oxytocin decreased the recognition accuracy for the natural micro-expressions of surprise and disgust. In summary, consistent with our hypothesis, the results of the current study showed that oxytocin may significantly impair the recognition of the micro-expressions of surprise and disgust.

In previous studies of macro-expressions, researchers found that oxytocin can significantly improve the recognition of macro-expressions of the six basic emotions (particularly for the macro-expressions of fear and happiness; Di Simplicio et al., 2009; Marsh et al., 2010; Leknes et al., 2012; Shahrestani et al., 2013; Fang et al., 2014; Leppanen et al., 2017; Shin et al., 2018; Schwaiger et al., 2019). However, previous studies on micro-expressions also found that some of the factors that are beneficial in the recognition of macro-expressions may actually be detrimental for the recognition of micro-expressions. For example, researchers found that while the facial mimicry was reported to beneficial for recognizing macro-expressions (for review, see Wood et al., 2016), facial mimicry of the lower face was actually found to be detrimental in the recognition of micro-expressions (Wu et al., 2016; Zeng et al., 2018). In addition, researchers also found that it was much easier to recognize the micro-expressions of outgroup members than the micro-expressions of ingroup members, which was completely opposite to the findings of the studies on macro-expressions (i.e., a ubiquitous pattern of ingroup advantage was found in the recognition of macro-expressions; Xie et al., 2019). Consistent with these researches, in the present research, we found that the neuropeptide oxytocin mainly plays an inhibiting role in the recognition of micro-expressions (especially for the micro-expression of surprise and disgust), which suggests that there are fundamental differences in the neurophysiological basis for the recognition of micro-expressions and macro-expressions. These results were consistent with the previous studies on the function of oxytocin, which showed that as a catalyst for social adaptation, the oxytocin may even have the power to make people more gullible. For example, researchers found that people under oxytocin treatment had more difficulties in predicting others’ decisions about whether to cooperate or defect (Israel et al., 2014); researchers also found that people under oxytocin would become particularly susceptible to lies told by people of the opposite sex (Pfundmair et al., 2017). The results of the current study were also consistent with the previous studies in which researchers found that some of the personality or emotional factors (e.g., emotion dysregulation, psychopathy, fearful attachment) that are associated with social maladjustment may actually promote the recognition accuracy for micro-expressions (Svetieva and Frank, 2016; Demetrioff et al., 2017; Felisberti, 2018). Given the close association between micro-expression and emotion hiding or deception (Ekman and Friesen, 1969; Ekman, 2003, 2009; Matsumoto and Hwang, 2011, 2018; Yan et al., 2013), the results of the present research further suggest that oxytocin may have the function of suppressing the processing of social signals that are associated with others’ untrustworthiness (such as micro-expressions) to promote social cohesions, which provide further support for the social adaptation account of oxytocin (Carter, 2014; Ma et al., 2016; Pfundmair et al., 2017; Xu et al., 2019).

It should be noted that although the current results suggest that the oxytocin may have emotion-specific effect on the recognition of micro-expressions, it is still possible that the nonsignificant effects of oxytocin on the recognition of angry and fearful micro-expressions might be caused by their low recognition performances within the present research (i.e., floor effect). More specifically, in previous researches, researchers already found that it was more difficult for untrained observers to recognize the fearful and angry micro-expressions than the micro-expressions of other emotions (e.g., Matsumoto and Hwang, 2011; Shen et al., 2012). In addition, in the present research, all our participants were inexperienced in recognizing micro-expressions (even in Study 3, they had only received the METT training for only once), which may further lead to the low recognition performances (i.e., the recognition accuracies were nearly at the chance level in the three studies) for these two kinds of micro-expressions. However, this floor effect can hardly explain why oxytocin did not affect the recognition of the micro-expressions of sadness and happiness in the present research since their recognition accuracies were much higher. Therefore, the nonsignificant results on micro-expressions of sadness and happiness were more likely to be caused by the emotion-specific effect of oxytocin. That is, since the oxytocin system may suppress the processing of signals that are related to deception to facilitate social adaptation (Ma et al., 2016; Pfundmair et al., 2017) and leaked surprise and disgust are more closely related to deception and other hostile intentions than the leaked emotion of sadness and happiness (e.g., Ten Brinke et al., 2012; Ten Brinke and Porter, 2012), it is possible for oxytocin to have a much more stronger inhibiting effect on the recognition of micro-expressions of surprise and disgust than the micro-expressions of sadness and happiness. To test external validity of the current results, researchers still need to further investigate the effects of oxytocin on the recognition of micro-expressions by employing more sufficient trainings and on other natural micro-expression datasets as well (e.g., the CAS(ME)^3^ natural micro-expression database; Li et al., 2022).

How does the oxytocin impair the recognition of micro-expressions of surprise and disgust? The answer may lie in the role of oxytocin in brain function. Previous studies on macro-expressions have suggested that intranasal oxytocin may affect the processing of the emotional information of surprise and disgust through its effect on the brain regions like insula and amygdala (e.g., Phillips et al., 1997; Kim et al., 2004; Fitzgerald et al., 2006; Scheele et al., 2014; Yao et al., 2018). This further suggests that the specific effects of oxytocin on the recognition of surprised and disgusted micro-expressions may be caused by the effects of oxytocin on insula and amygdala. In fact, previous research on micro-expressions has already found that the recognition accuracy of micro-expressions could be predicted by the resting-state brain activities of insula and amygdala (Zhang et al., 2020a). Previous studies also found that oxytocin can affect the facial mimicry process (Korb et al., 2016; Pavarini et al., 2019) and the facial mimicry was found to exert its effect on emotion processing through its effect on the neural responses of insula and amygdala (e.g., Lee et al., 2006; Rymarczyk et al., 2019). Moreover, intranasal oxytocin was found to attenuate the neural responses to the subliminal or near subliminal presented negative facial expressions (e.g., fear, anger) within the regions of insula and amygdala (Kanat et al., 2015; Luo et al., 2017). Considering that these subliminal or near subliminal presented facial expressions may share some common features with standardized micro-expressions (i.e., short presentation duration or backward masking; Matsumoto et al., 2000), these studies further suggest that the modulation of the neural activities of insula and amygdala might be the neuropsychological mechanism for the inhibiting effects of oxytocin on micro-expression recognition. However, it should be noted that researches also showed that at the behavioral level, intranasal oxytocin can promote the sensitivity to subliminal or near subliminal presented facial expressions (Schulze et al., 2011; Leknes et al., 2012). Given that the results of these two studies (Schulze et al., 2011; Leknes et al., 2012) were opposite to the results of the present research and there were significant differences in the facial dynamics between these subliminal or near subliminal presented facial expressions and micro-expressions (Ekman, 2003; Porter and ten Brinke, 2008; Yan et al., 2013; Matsumoto and Hwang, 2018; Zeng et al., 2018), the results of the current study also suggest that the exact role of oxytocin in emotion perception may depend on the specific facial dynamics of facial expressions. This account can also explain the slight discrepancies among the results of the three studies of the present research (i.e., the oxytocin impaired the recognition speed for surprised micro-expressions only under 150 ms in Study 1, but it decreased the recognition accuracy for disgusted micro-expressions in Study 2 and impaired the recognition for both surprised and disgusted micro-expressions in Study 3). That is, the specific effects of oxytocin on micro-expression recognition were modulated by the specific facial dynamics of micro-expressions (there were significant differences in facial dynamics among the standardized intense, subtle and natural micro-expressions; Yan et al., 2013; Zeng et al., 2018). The psychological and brain mechanisms for the emotion-specific effects of oxytocin on micro-expression recognition demands investigation in the future.

Following previous studies (e.g., Leknes et al., 2012; Shin et al., 2018), in the present research we employed a setting of total dose of 40 IU intranasal oxytocin to investigate the effects of oxytocin on micro-expression recognition. However, other different settings of dosage of oxytocin (e.g., 16 IU, 20 IU, 24 IU, 30 IU) were also employed in the studies of macro-expressions (for review, see Romero-Martínez et al., 2021). Although previous studies on macro-expressions have mainly employed the dosage of 24 IU (Romero-Martínez et al., 2021), previous research also showed that there were no significant differences between the effects of 24 IU oxytocin and 40 IU oxytocin on social behaviors (Zhao et al., 2017). However, it is still possible that the emotion-specific effects of oxytocin we found in the present research may depend on the specific setting of the dosage in the current research. Therefore, we still need to investigate whether the dosage of oxytocin can moderate the effects of intranasal oxytocin on micro-expression recognition in the future. In addition, we should note that following previous studies (Di Simplicio et al., 2009; Prehn et al., 2013; Fang et al., 2014; Feeser et al., 2014; Korb et al., 2016; Shin et al., 2018), in the present research we employed males-only samples. Although this choice has some merits for practical reasons (e.g., reducing complications linked to female menstrual cycle; Di Simplicio et al., 2009; Prehn et al., 2013; Fang et al., 2014; Feeser et al., 2014; Korb et al., 2016; Shin et al., 2018), previous study has also suggested that the effects of oxytocin on emotion perception may differ between sexes (Luo et al., 2017). Therefore, we still need to investigate whether the effects of oxytocin on micro-expression recognition are moderated by participants’ gender in the future.

Previous study has suggested that the recognition process for the micro-expressions of ingroup members may be different from the recognition process of the micro-expressions of outgroup members (Xie et al., 2019). This further suggests that the effects of oxytocin may depend on the group membership of the expresser of micro-expressions. However, in the present research, the models of micro-expressions were randomly selected, which made the numbers of ingroup and outgroup targets were unmatched (particularly for Study 1 and Study 2). In addition, we also did not match the recognition accuracy for the macro-expressions of these selected models as in previous study (Xie et al., 2019), which also made the comparison between the recognition accuracies of ingroup and outgroup members invalid for the present study. Given that previous studies have already showed that the effects of oxytocin may differ between the ingroup and outgroup targets (e.g., De Dreu et al., 2011), it is important for researchers to investigate whether the effects of oxytocin on micro-expression recognition can be moderated by the group membership of the expressers in the future.

## Conclusion

By employing a standard protocol of single dose of intranasally administrated oxytocin, the present research is the first to demonstrate that the oxytocin system has participated in the processing of micro-expressions. It finds that intranasal oxytocin can specifically decrease the recognition accuracy or the recognition speed for micro-expressions of surprise and disgust, which suggest that oxytocin mainly plays an inhibiting role in the recognition of micro-expressions.

## Data Availability Statement

The raw data supporting the conclusions of this article will be made available by the authors, without undue reservation.

## Ethics Statement

The studies involving human participants were reviewed and approved by Research Ethics Committee of Hunan Normal University. The patients/participants provided their written informed consent to participate in this study. Written informed consent was obtained from the individual(s) for the publication of any potentially identifiable images or data included in this article.

## Author Contributions

QW and YX conceived and designed the studies. QW, YX, XL, and YL performed the studies. QW, YX, and XL analyzed the data. QW drafted the paper. All authors contributed to the article and approved the submitted version.

## Funding

This work was supported by the Outstanding Young Scientific Research Project of Hunan Provincial Department of Education (19B361).

## Conflict of Interest

The authors declare that the research was conducted in the absence of any commercial or financial relationships that could be construed as a potential conflict of interest.

## Publisher’s Note

All claims expressed in this article are solely those of the authors and do not necessarily represent those of their affiliated organizations, or those of the publisher, the editors and the reviewers. Any product that may be evaluated in this article, or claim that may be made by its manufacturer, is not guaranteed or endorsed by the publisher.

## Footnotes

^1^Intranasally administrated oxytocin travels to the brain *via* olfactory and trigeminal nerve fibers (Quintana et al., 2021).

^2^Following previous studies (Wu et al., 2016; Zeng et al., 2018), we also called the micro-expressions that are high in the intensity of facial actions as intense micro-expressions, and called the micro-expressions that are low in intensity as subtle micro-expressions.

^3^Due to copyright issues, the facial expression databases and the micro-expression recognition training tool employed in the present research are not provided. Seek permissions from their original authors to obtain these materials.

^4^Therefore, the micro-expressions that were presented by the JACBART paradigm are referred as standardized micro-expressions in the present research.

^5^Micro-expressions of some emotions can hardly be elicited, therefore in the natural micro-expression databases, the number of micro-expressions within different emotion categories were not equal (Yan et al., 2013, 2014; Yap et al., 2020).
